# A rare case of seven siblings with Waardenburg syndrome: a case report

**DOI:** 10.1186/s13256-018-1704-1

**Published:** 2018-07-05

**Authors:** Luma Haj Kassem, Mohamed Fares Ahmado, Majd Sheikh Alganameh

**Affiliations:** ENT Department, Aleppo University Hospital, Aleppo, Syria

**Keywords:** Waardenburg syndrome, Type 2, Siblings, Hearing loss, Heterochromia, Mutation De novo, Case report

## Abstract

**Background:**

Waardenburg syndrome is a group of rare genetic conditions. It is determined by the absence of melanocytes from the eyes, hair, and skin. There are four types of Waardenburg syndrome with specific criteria to diagnosis the different types. The main clinical manifestations are facial abnormalities, pigmentary defects, and hearing loss with no specific predilection with regard to sex or race.

**Case presentation:**

An Arabic Syrian family, consisting of 14 siblings from third-degree relative parents with a low income, living in the Syrian countryside, presented to our institute with their 8-year-old son who had congenital hearing loss that had led to his inability to speak.

He has six siblings who had congenital sensory hearing loss proven by auditory brainstem response tests at an early age. An otoacoustic emissions test and a pure-tone audiogram were performed for our patient and showed sensory hearing loss.

An interesting feature in the last seven siblings was that some of them have heterochromia iris, and the others have segmental heterochromia in their iris.

An ophthalmology consultation was performed to detect any other features or disorders. A dermatology consultation, laboratory tests, and chest X-ray were also performed for all the siblings and revealed no abnormalities. There was no history for musculoskeletal system or intestinal disorders. Based on the Waardenburg criteria, our patient and his six siblings all have Waarenburg syndrome.

**Conclusions:**

Although the inheritance of Waardenburg syndrome is autosomal dominant, de novo cases of this rare syndrome are mentioned in the medical literature. We report a unique case of seven siblings with Waardenburg syndrome. This case report shows the crucial role of consanguineous parents on this syndrome, and indicates that the number of children with this rare syndrome is increasing.

## Background

Waardenburg syndrome (WS) is a rare inherited disorder [1]. WS is characterized by deafness in association with pigmentary anomalies and various defects of neural crest-derived tissues [1, 2]. The incidence of WS is estimated at 2/100000 worldwide. The international distribution of this disease can be observed without preference for race or gender [3].

WS may be diagnosed at birth or in early childhood or, in some cases, at a later age based upon a thorough clinical evaluation, identification of characteristic physical findings, a complete patient and family history, and various specialized studies [4].

We report here a case of seven siblings with WS and without family history of the disorder.

## Case presentation

An Arabic Syrian family consisting of 14 siblings and their parents, who live in the Syrian countryside, presented to our Ear, Nose, Throat (ENT) department at Aleppo University Hospital with their 8-year-old son to get a medical report for his hearing disability at the request of a social association.

Our patient has congenital hearing loss, which had led to his inability to speak. His medical history was unremarkable, but his family history was surprising. His parents are third-degree relatives and he has six siblings who have the same condition. There is a lack of communication among the siblings and their family.

All six siblings had been diagnosed by auditory brainstem response (ABR) test, since this test was available. All the siblings were full-term babies and the mother denied any problems during pregnancy or in the postnatal period.

We had asked our patient’s parents to attend again with all seven children in order to examine all of them.

At our ENT department, an ear examination performed by otoscope showed normal findings. A tuning fork test, otoacoustic emissions (OAEs), and a pure-tone audiogram (PTA) revealed that the seven siblings have bilateral sensorineural hearing loss (SNHL).

**Fig. 1:**
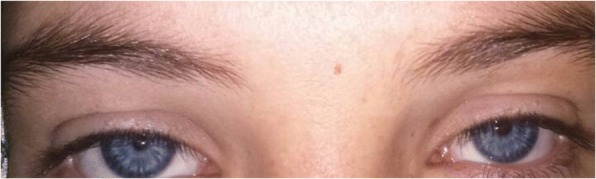
Pale blue eyes

**Fig. 2:**
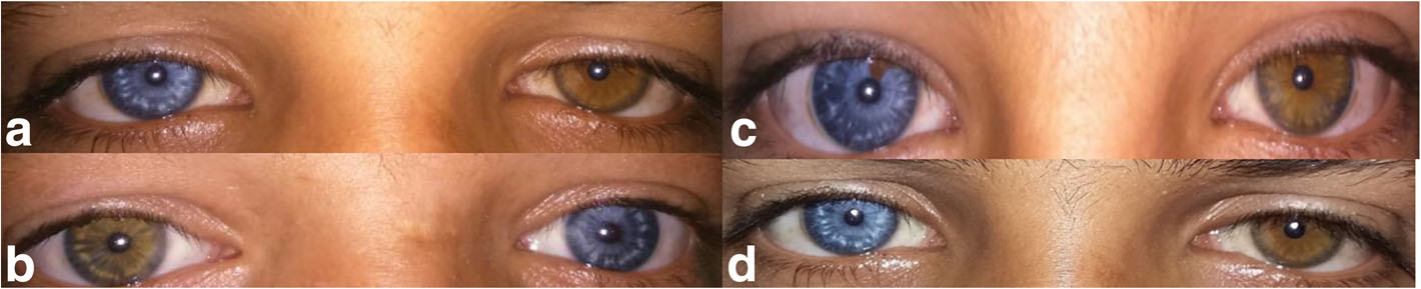
**a**, **b**, **c**, **d** A full heterochromia of the four siblings

**Fig. 3:**
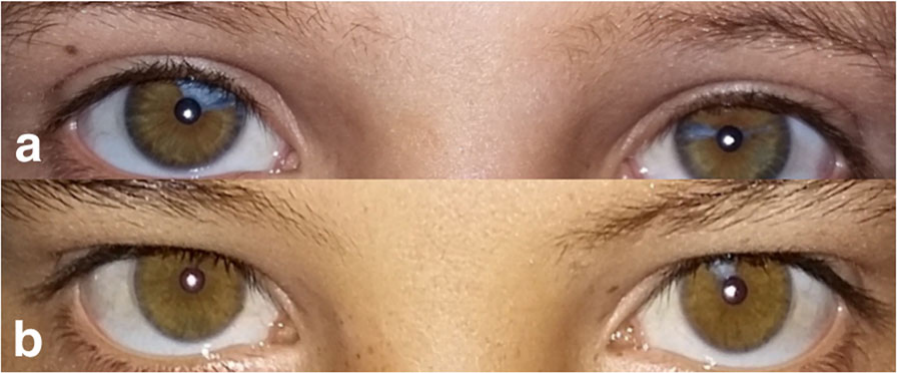
Segmental pigmentation in the iris of (**a**) our patient (**b**) the iris of a sibling

On inspection, we also found that all seven siblings have a prominent nose root. We noticed that the iris colors of the seven siblings are distributed into three distinguished types. One of them has pale blue eyes (Fig. 1); four have full heterochromia (Fig. 2), and the other two have a segmental pigmentation in their iris (Fig. 3).

An ophthalmology consultation for all the seven siblings was performed and reve**a**led that they do not have any abnormalities. An examination by a slit lamp (except for the colors of their iris) was unremarkable. An ocular motility test, a color vision, and a dilated fundus examination by fundscopy were all normal.

A dermatology consultation, another consultation to look for musculoskeletal system or cranial skeletal abnormalities, laboratory tests (such as blood tests and urine analysis), and chest X-ray were also performed for all the siblings and revealed no abnormities.

**Fig. 4:**
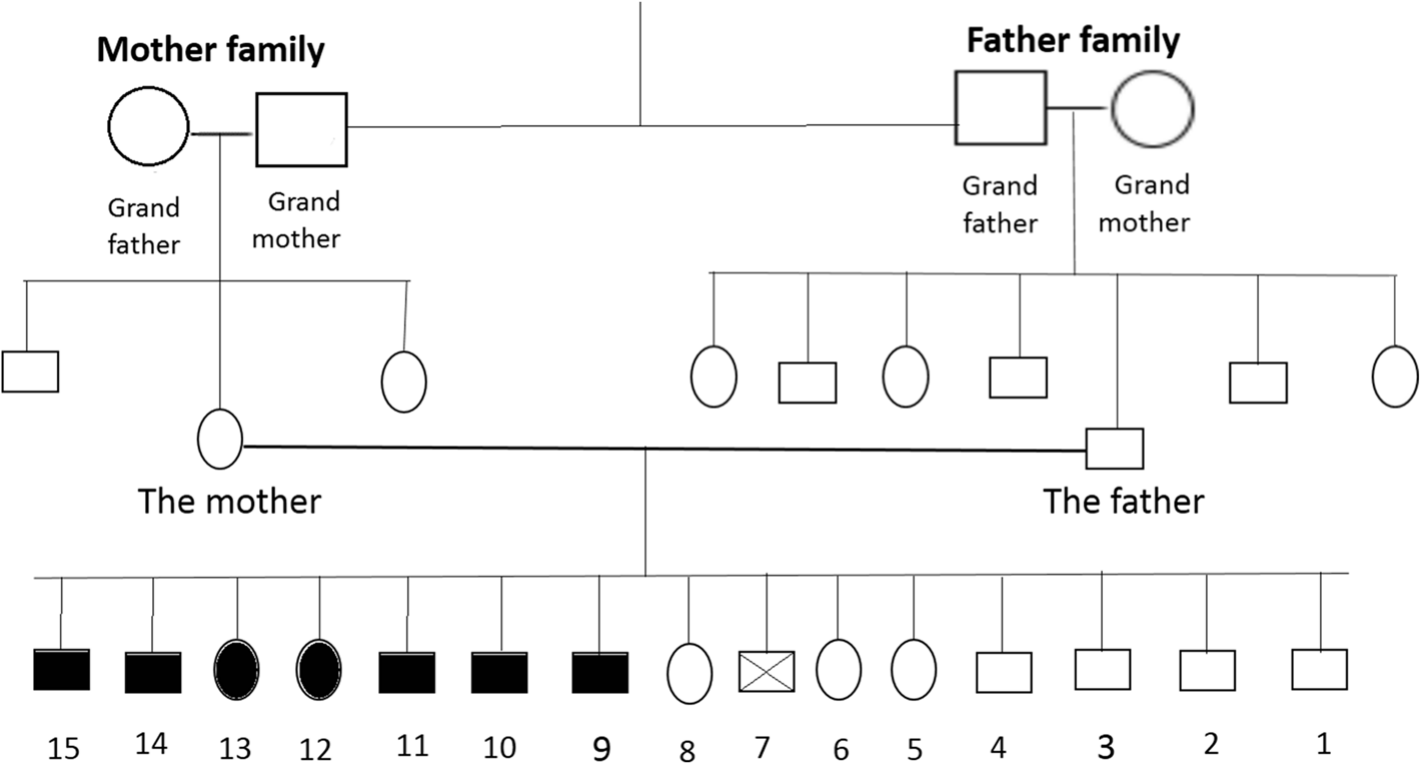
A concise pedigree chart of three generations of this family. Due to space constraints, no cousins were added to this table. An important point is that the parents are cousins. All eight children are totally normal; however, the seventh child died by the age of 1

We collected data on all seven siblings with their ages, gender, physical examination, investigations and rehabilitation details (Table 1), and a concise pedigree chart of three generations of this family (Fig. 4).

**Table 1:** A table for the seven siblings with their ages, gender, physical examination, investigations, and rehabilitation details

Child the 7 siblings	Gender	Age	Symptoms and physical examination		Investigations	Rehabilitation
1	Male	20-year-old		Congenital sensory neural hearing loss + Prominent broad nasal root	Ear examination (normal findings) + Otoacoustic emissions (OAEs) + ABR at early age + Dermatology consultation + Laboratory tests (blood and urine analysis) + Chest X-ray + Opthalmology consultation (an examination by a slit lamp, color vision, ocular motility, and fundoscopy)	Ability to read lips + Speech therapist is consulted again
2	Male	17-year-old				
3	Male	15-year-old	Full heterochromia			
4	Female	14-year-old				
5	Female	13-year-old	Pale blue eyes			
6	Male	11-year-old	Segmental pigmentation			
7 (Our patient)	Male	8-year-old	Congenital sensory neural hearing loss + Prominent broad nasal root + Segmental pigmentation in the iris		Ear examination (normal findings) + Otoacoustic emissions (OAEs) + Pure-tone audiogram (PTA) + Dermatology consultation + Laboratory tests (blood and urine analysis) + Chest X-ray + Opthalmology consultation (an examination by a slit lamp, color vision, ocular motility, and fundoscopy)	Speech therapist is consulted and communication skills sessions

Only one cousin of our patients has Down syndrome; no mental disabilities are found in the history of this family.

Although a congenital SNHL diagnosis for the six siblings was made at an early age, unfortunately no artificial cochlear implantation was performed or hearing aid provided.

The family could not commit to attending the rehabilitation center and the communication skills sessions because of the distance from their home (they live in the countryside), their poor financial circumstances, and the 6 years of war they had lived though. The seven siblings have the ability to read lips; however, a speech therapist was consulted after their last visit.

## Discussion

Petrus Johannes Waardenburg, a Dutch ophthalmologist, was the first to describe the rare inherited disorder in 1951 [1]. It affects approximately 1:40,000 of the population and comprises 3% of congenitally deaf children. It has no racial or ethnic predilection and has an equal male to female ratio [2].

To diagnose WS**,** five major and five minor diagnostic criteria have to be established. The major criteria include sensorineural hearing loss, a white forelock, pigmentary disturbance of the iris, dystopia canthorum (lateral displacement of the inner eye corners), and first-degree relatives diagnosed with WS. Minor criteria include congenitally hypopigmentation of the skin, medial eyebrow flare (synophrys), hypoplastic alae nasi, prominent broad nasal root, and early graying of hair before the age of 30. The clinical diagnosis of WS requires at least two major criteria or one major and two minor criteria [1–6].

According to the diagnostic criteria proposed by Waardenburg, there are three features of major criteria in this case: sensory neural hearing loss (SNHL), abnormal pigmentation of the iris, and diagnosed first-degree relatives. There is also one minor feature, that is, prominent broad nasal root.

WS is a full clinical picture, that is, every case is unique in its features. The differential diagnosis of WS is in accordance with the main features in our patient. In fact, the congenital SNHL was the main issue for our patient and his six siblings. The other criteria complete the clinical picture, but they are not the main problems that affect their lives.

Some individuals with WS are affected by congenital deafness. Such hearing impairment appears to result from abnormalities or absence of the organ of Corti [3–5]. Hearing loss in WS has a congenital, sensorineural character, and is usually non-progressive, varying from slight to profound [3–5].

In most affected individuals with WS, congenital SNHL is in both ears. However, in rare cases, only one side may be affected. Evidence suggests that congenital SNHL is more frequently associated with WS2 than WS1 [1, 3–6].

In our case, all six siblings have bilateral congenital profound sensorineural hearing loss which was proven by auditory brainstem response test (ABR), since this test was available. By contrast, with our patient, we could not perform an ABR for him, whereas an ear examination by otoscope showed normal structures and an (OAEs and a PTA were performed and revealed a deep sensory hearing loss. A hearing aid is required as well as a speech therapist consultation.

Some reports suggest that heterochromia irides may be more frequent in WS2. The affected individuals may have unusually pale blue eyes or differences in the pigmentation of the two irides or within different areas of the same iris (heterochromia irides) [4]. The bilateral blue iris is the most prominent feature in these patients. Iris heterochromia may be complete or partible.

In complete heterochromia each iris is a different color, while in partial heterochromia the differently colored area of the iris is sharply demarcated from the remainder and is usually, but not invariably, a radial segment. Partial heterochromia may be unilateral or bilateral and, if bilateral, may be symmetrical or asymmetrical [3–5].

An interesting aspect is that the three types of pigmentary disturbances of iris are found in our case. (a) Complete heterochromia iridium: two eyes of different color (Fig. 2), (b) partial or segmental heterochromia: segments of blue or brown pigmentation in one eye (our patient) (Fig. 3), (c) pale blue eyes: characteristic brilliant blue in both eyes (Fig. 1). A regular ophthalmic examination is recommended to follow up any probable lesions.

Hypopigmentation of the skin is congenital and may be found on the face, trunk, or limbs. It may be associated with an adjacent white forelock, which can present before the age of 30 years. Hyperpigmentation has also been described. Pigmentation defects can affect the eyebrows and eyelashes as well as scalp hair [5].

In the history of the six siblings there was a dermatology consultation. A new consultation for them as well our patient revealed no skin or hair lesions.

The four types of WS are: type 1 and type 2 are the most common and often similar in clinical manifestation [1]. Clinically, we can distinguish between those two types only by dystopia canthorum, which is characteristic of WS type 1 (WS1) and absent in WS type 2 (WS2) [3].

The Waardenburg (W) index is a biometric index based on interpupillary, inner canthi, and outer canthal distances. A W index larger than 1.95 is considered positive for dystopia canthorum [1]. The W index for seven siblings was less than 1.95, which can exclude the probability of type 1.

Type 3 has the same symptoms as type 1 with musculoskeletal limb abnormalities. It may manifest with a cleft palate, musculoskeletal system hypoplasia, and syndactly [3].

Type 4 is called Shah–Waardenburg syndrome and is associated with Hirschsprung disease, its most defining feature being the aganglionic megacolon [3]. The seven siblings did not have any history of musculoskeletal or intestinal disorders especially during the neonatal period.

Inheritance of Waardenburg syndrome is autosomal dominant, with the majority of the probands with affected parents. A minority does not have an affected parent, and may be presumed to be a de novo case [2].

We can interpret the incidence of such a rare disease in this family as the researchers indicate that the disorder may sometimes result spontaneously for unknown reasons from mutations in some individuals with WS1 or WS with no apparent family history of the disorder [4].

## Conclusions

Unfortunately, we could not have done genetic counseling previously due to financial constraints and the genetic tests are still unavailable until the time of writing.

Consanguineous marriage and having a large number of children could be a crucial factor in the occurrence of this syndrome.

## Key Messages

Waardenburg syndrome is a rare disease and its inheritance is autosomal dominant, but we should consider de novo cases of this rare syndrome, especially in families with a large number of children. We report this case to spread awareness of the important role of consanguineous marriage in Waardenburg syndrome and highlight that the number of children with this rare syndrome is increasing.

## Abbreviations

ABR: Auditory brainstem response test
OAEs: Otoacoustic emissions
PTA: Pure-tone audiogram
SNHL: Sensory neural hearing loss
WS: Waardenburg syndrome
WS1/2: Waardenburg syndrome type 1/2
